# ASO Author Reflections: Perioperative Lymphopenia Following Hepatectomy for Hepatocellular Carcinoma: A Routine Yet Overlooked Prognostic Biomarker

**DOI:** 10.1245/s10434-023-14856-8

**Published:** 2024-01-04

**Authors:** Diamantis I. Tsilimigras, Timothy M. Pawlik

**Affiliations:** Department of Surgery, Division of Surgical Oncology, James Comprehensive Cancer Center, The Ohio State University Wexner Medical Center, Columbus, OH USA

## Past

Immune dysregulation plays an important role in cancer progression.^1,2^ Lymphopenia, defined as the absence or reduced count of host lymphocytes, in the perioperative period might indicate a state of pre-existing cancer-related immune tolerance.^3,4^ Although laboratory tests are routinely obtained prior to and following a hepatectomy for hepatocellular carcinoma (HCC), little is known about the incidence and clinical significance of perioperative lymphopenia among patients undergoing resection for HCC. In this study, we sought to investigate whether preoperative or surgical stress-induced lymphopenia (i.e. absolute lymphocyte count [ALC] <1000/μL) was predictive of short- and long-term outcomes following HCC resection.^5^

## Present

In this international, multi-institutional study, a total of 1448 patients underwent resection of HCC between 2000 and 2020. Median preoperative ALC was 1593/μL (interquartile range [IQR] 1208–2006). The incidence of preoperative lymphopenia was 14.0%, whereas postoperative day (POD) 1, POD3 and POD5 lymphopenia rates were 50.2%, 45.1% and 35.6%, respectively. Preoperative lymphopenia was associated with worse 5-year overall survival (OS), cancer-specific survival (CSS) and disease-free survival (DFS), as was lymphopenia on POD1, POD3 and POD5. Patients with severe lymphopenia (ALC <500/μL) on POD5 had worse OS and DFS (5-year OS: 44.7 vs. 54.3% vs. 66.1%; 5-year DFS: 27.8% vs. 33.3% vs. 42.3%; both *p* < 0.05) as well as higher incidence of overall (45.5 vs. 25.3% vs. 30.9%; *p* = 0.013) and major complications (18.2 vs. 3.4 vs. 4.5%; *p* < 0.001) versus individuals with moderate (ALC 500–1000/μL) or no lymphopenia following hepatectomy for HCC. While persistent lymphopenia on POD5 was not associated with worse long-term outcomes among patients with known, preoperative lymphopenia, new-onset lymphopenia on POD5 predicted worse OS among individuals with normal ALC prior to surgery (57.8 vs. 66.9%; *p* = 0.028). On multivariable analysis, prolonged lymphopenia on POD5 was independently associated with higher hazards of death (hazard ratio [HR] 1.38, 95% confidence interval [CI] 1.11–1.72) and recurrence (HR 1.22, 95% CI 1.02–1.45).

## Future

Data from the current study demonstrated that perioperative lymphopenia may predict short- and long-term outcomes among patients undergoing curative-intent resection for HCC. Severity of lymphopenia on POD5 can sub-stratify patients relative to long-term outcomes after HCC resection. Evaluation of ALC in the perioperative period, although frequently overlooked, should be considered as an important prognostic factor among individuals undergoing hepatectomy for HCC.
